# Convergent and sequential synthesis designs: implications for conducting and reporting systematic reviews of qualitative and quantitative evidence

**DOI:** 10.1186/s13643-017-0454-2

**Published:** 2017-03-23

**Authors:** Quan Nha Hong, Pierre Pluye, Mathieu Bujold, Maggy Wassef

**Affiliations:** 1 Department of Family Medicine, McGill University, 5858 Chemin de la Côte-des-Neiges, 3rd Floor, Montreal, QC H3S 1Z1 Canada; 2 Information Technology Primary Care Research Group, Department of Family Medicine, McGill University, Montreal, QC Canada

**Keywords:** Systematic review, Research synthesis, Mixed studies review, Mixed methods review, Integrative review, Mixed methods research

## Abstract

**Background:**

Systematic reviews of qualitative and quantitative evidence can provide a rich understanding of complex phenomena. This type of review is increasingly popular, has been used to provide a landscape of existing knowledge, and addresses the types of questions not usually covered in reviews relying solely on either quantitative or qualitative evidence. Although several typologies of synthesis designs have been developed, none have been tested on a large sample of reviews. The aim of this review of reviews was to identify and develop a typology of synthesis designs and methods that have been used and to propose strategies for synthesizing qualitative and quantitative evidence.

**Methods:**

A review of systematic reviews combining qualitative and quantitative evidence was performed. Six databases were searched from inception to December 2014. Reviews were included if they were systematic reviews combining qualitative and quantitative evidence. The included reviews were analyzed according to three concepts of synthesis processes: (a) synthesis methods, (b) sequence of data synthesis, and (c) integration of data and synthesis results.

**Results:**

A total of 459 reviews were included. The analysis of this literature highlighted a lack of transparency in reporting how evidence was synthesized and a lack of consistency in the terminology used. Two main types of synthesis designs were identified: convergent and sequential synthesis designs. Within the convergent synthesis design, three subtypes were found: (a) data-based convergent synthesis design, where qualitative and quantitative evidence is analyzed together using the same synthesis method, (b) results-based convergent synthesis design, where qualitative and quantitative evidence is analyzed separately using different synthesis methods and results of both syntheses are integrated during a final synthesis, and (c) parallel-results convergent synthesis design consisting of independent syntheses of qualitative and quantitative evidence and an interpretation of the results in the discussion.

**Conclusions:**

Performing systematic reviews of qualitative and quantitative evidence is challenging because of the multiple synthesis options. The findings provide guidance on how to combine qualitative and quantitative evidence. Also, recommendations are made to improve the conducting and reporting of this type of review.

## Background

Systematic reviews have been used by policy-makers, researchers, and health service providers to inform decision-making [1]. Traditionally, systematic reviews have given preference to quantitative evidence (mainly from randomized controlled trials (RCTs) and to clinical effectiveness questions). However, a focus on quantitative evidence is insufficient in areas where research is not dominated by RCTs [2]. For example, in several fields such as public health, RCTs are not always appropriate nor sufficient to address complex and multifaceted problems [3]. Also, while reviews focusing on RCTs can help to answer the question, “What works for whom?,” other important questions remain unanswered such as “Why does it work?,” “How does it work?,” or “What works for whom in what context?.” Such questions can be addressed by reviewing qualitative evidence. Indeed, the analysis of qualitative evidence can complement those of quantitative studies by providing better understanding of the impact of contextual factors, helping to focus on outcomes that are important for patients, families, caregivers, and the population and exploring the diversity of effects across studies [4].

In recent years, there has been a growing interest in synthesizing evidence derived from studies of different designs. This new type of review has been labelled with various terms such as integrative review [5], mixed methods review [6], mixed methods research synthesis [7], mixed research synthesis [8], and mixed studies review [9, 10]. These reviews can yield a rich and highly practical understanding of complex interventions and programs [9, 10]. They can be used to provide (a) a deeper understanding of quantitative evidence, (b) a statistical generalization of findings from qualitative evidence, or (c) a corroboration of knowledge obtained from quantitative and qualitative evidence [9].

The past decade has been rich with methodological advancements of reviews of qualitative and quantitative evidence. For example, several critical appraisal tools for assessing the quality of quantitative and qualitative studies have been developed [9, 11, 12]. Also, new synthesis methods have been developed to integrate qualitative and quantitative evidence such as critical interpretive synthesis, meta-narrative synthesis, and realist synthesis [4, 13, 14]. In addition, researchers have been interested in defining and categorizing different types of synthesis designs (see Table 1). These types were inspired by the literature on mixed methods research, which is a research process integrating quantitative and qualitative methods of data collection and analysis [15]. The types of synthesis design developed are, as yet, theoretical; they have not been tested on a large sample of reviews. Therefore, it is necessary to gain a better understanding of how reviews of qualitative and quantitative evidence are carried out. The aim of this review of reviews was to identify and develop a typology of synthesis designs and methods and to propose strategies for synthesizing qualitative and quantitative evidence.

**Table 1:** Typology of synthesis designs suggested in the literature

Authors	Synthesis designs^a^
Frantzen and Fetters [40]	1. Convergent meta-integration: quantitative, qualitative, and mixed methods studies are synthesized without data transformation. 2. Convergent qualitative meta-integration: quantitative data are transformed into qualitative format. 3. Convergent quantitative meta-integration: qualitative data are transformed into quantitative format. Each design can be of basic type (when a review includes quantitative and qualitative studies) or advanced type (when a review includes qualitative, quantitative, and mixed methods studies).
Heyvaert et al. [22]	An 18-design framework based on the emphasis of approaches (equal or dominant status of qualitative or quantitative approach), the temporal orientation (sequential or convergent), and the level of integration (partial or full integration).
Pluye and Hong [10]	1. Sequential exploratory: results of the qualitative synthesis inform the quantitative synthesis. 2. Sequential explanatory: results of the quantitative synthesis inform the qualitative synthesis. 3. Convergent: results of qualitative and quantitative studies are integrated using data transformation techniques.
Sandelowski et al. [8]	1. Segregated: qualitative and quantitative findings are treated separately. 2. Integrated: qualitative findings are transformed into quantitative data (quantitizing) or quantitative finding are transformed into qualitative data (qualitizing). 3. Contingent: cycle of research synthesis studies conducted to answer questions raised by previous synthesis.

^a^These synthesis designs are theoretical and not tested on a large sample of reviews

This review of reviews will contribute to a better understanding of the extent of this literature and justify its relevance. The results will also provide a comprehensive roadmap on how reviews of qualitative and quantitative evidence are carried out. It will provide guidance for conducting and reporting this type of review.

## Methods

A review of systematic reviews combining qualitative and quantitative evidence (hereafter, systematic mixed studies reviews (SMSR)) was performed (Table 2). SMSR follows the typical stages of systematic review, with the particularity of including evidence from qualitative, quantitative, and/or mixed method studies [7, 10]. It uses a mixed methods approach [7, 10].

**Table 2:** Three levels of research

	Level of research		
	Primary	Secondary^a^	Tertiary
Research	Empirical study: research based directly on observation, experiment, or simulation rather than on reasoning or theory alone [26, 47].	Systematic review: collation and interpretation of existing empirical studies using systematic and explicit methods [48].	Review of reviews: collation and interpretation of existing systematic reviews [48].
Types of research	Qualitative study: research that aims at exploring and understanding phenomena in terms of the meanings people bring to them [49, 50].	Qualitative review: review combining qualitative studies.	Review of qualitative reviews: review combining qualitative reviews.
	Quantitative study: research that aims at testing theories by examining the relationship among variables [49].	Quantitative review: review combining quantitative studies.	Review of quantitative reviews: review combining quantitative reviews.
	Mixed methods study: research involving collecting and integrating both quantitative and qualitative data [49].	Mixed studies review: review combining qualitative, quantitative, and/or mixed methods studies.	Review of mixed studies reviews: review combining mixed studies reviews.
Data	Primary data collected from fieldwork or lab work.	Findings from included studies.	Findings from included reviews.
Data analysis	Analysis: a step within empirical study of investigating, making sense of, interpreting, and/or theorizing primary data using statistical and/or text analysis procedures [49, 51].	Synthesis: a step within a systematic review consisting of creating something new of findings from included studies [48].	Synthesis of findings across included reviews.

^a^Secondary research is different from secondary analysis. Secondary analysis is used to designate the reanalysis of primary data to answer new questions [52]

The focus of this review of reviews was on the synthesis process that is the sequence of events and activities regarding how the findings of the included studies were brought together. Thus, a “process-data conceptualization” was conducted [16] using a deductive-inductive approach, i.e., using concepts from the literature on mixed methods research as a starting point, but allowing for new concepts to emerge. Based on the literature on mixed methods research, three main questions were asked: (a) Was the evidence synthesized using qualitative and/or quantitative synthesis methods?, (b) Was there a sequence in the synthesis of the evidence?, and (c) Where did the integration of quantitative and qualitative evidence occur?

### Information sources and search strategy

Reviews were searched in six databases (Medline, PsycInfo, Embase, CINAHL, AMED, and Web of Science) from their respective inception dates through December 8, 2014. A search strategy was developed by the first author with the help of two specialized librarians. It included only free text searching since the field of SMSR is still new and no controlled vocabulary exists (see Table 3 for full-search strategy in Medline). All the records were transferred to a reference manager software (EndNote X7) and duplicates were removed using the Bramer-method [17].

**Table 3:** Search strategy (in Medline)

Concepts	Terms searched
Mixing studies, methods, or data	1. mixed method*.mp 2. mixed stud*.mp 3. mixed research.mp 4. mixed knowledge.mp 5. multi-method*.mp 6. multimethod* 7. multiple method*.mp 8. OR/1-7
Quantitative and qualitative	9. quantitative.mp 10. trial*.mp 11. qualitative.mp 12. 9 or 10 13. 11 and 12
Reviews or syntheses	14. systemat* review*.mp 15. systemat* synthes*.mp 16. critical review*.mp 17. critical synthes*.mp 18. structured review*.mp 19. structured synthes*.mp 20. integrat* review*.mp 21. integrat* synthes*.mp 22. (literature adj3 review*).mp 23. (literature adj3 synthes*).mp 24. research review*.mp 25. research synthes*.mp 26. evidence review*.mp 27. evidence synthes*.mp 28. comprehensive review*.mp 29. comprehensive synthes*.mp 30. OR/14-29
Specific synthesis methods	31. realist review*.mp 32. realist synthes*.mp 33. meta-narrative review*.mp 34. meta-narrative synthes*.mp 35. critical interpretive review*.mp 36. critical interpretive synthes*.mp 37. 31 or 32 or 33 or 34 or 35 or 36
Combination and limits	38. 8 or 13 39. 30 and 38 40. 37 or 39 41. limit 40 to (English or French)

### Eligibility criteria and selection

SMSRs were included in this review of reviews if they provided a clear description of search and selection strategies, a quality appraisal of included studies, and combined either (a) qualitative, quantitative, and/or mixed methods studies; (b) qualitative and mixed methods studies; (c) quantitative and mixed methods studies; or (d) only mixed methods studies. However, reviews that combined qualitative and mixed methods studies but only analyzed the qualitative evidence of the mixed methods studies were excluded. Likewise, reviews that included quantitative and mixed methods studies but only analyzed quantitative evidence were excluded. SMSRs limited to bibliometric analysis, as well as those that contained only a secondary analysis of studies from previous systematic reviews, were excluded. Also, reviews not published in English or French were excluded.

A three-step selection process was followed. First, all publications that were not journal papers were excluded in EndNote. Second, the remaining records were transferred to the DistillerSR software and two reviewers independently screened all the bibliographic records (titles and abstracts). When the two reviewers disagreed regarding the inclusion/exclusion of a bibliographic record, it was retained for further scrutiny at the next step. Third, two independent reviewers read the full texts of the potentially eligible reviews. Reviews for which the type of studies was not clear (e.g., no description of included studies) were excluded. Also, some reviews were excluded during the analysis because they considered quantitative surveys as qualitative studies. Disagreements were reconciled through discussion or arbitration by a third reviewer.

### Data collection and synthesis

One reviewer extracted the following data using NVivo 10: year, country, number of included studies, review title, justification for combining qualitative and quantitative evidence, and synthesis methods mentioned.

The quality of the retained reviews was not critically appraised because the aim of this review of reviews was to have a better understanding of how the synthesis is performed in SMSRs. In general, performing an appraisal is useful to check the trustworthiness of individual studies to a review and if the quality might impact the review findings [18]. This review of reviews did not focus on the findings of each review but put emphasis on the synthesis method used and how the findings were presented. Also, while some tools for appraising systematic reviews of quantitative studies exist [19, 20], to our knowledge, there is no tool for appraising the quality of SMSRs.

The data describing the synthesis processes of included reviews were analyzed using the visual mapping technique, which is commonly used for conceptualizing process data [16]. Two reviewers created visual diagrams to represent the synthesis process, i.e., the means by which the qualitative and quantitative evidence, synthesis methods, and findings were linked. These diagrams were then compared and categorized into ideal types. An ideal type is defined as the grouping of characteristics that are common to most cases of a given phenomenon [21].

The analysis focused on three concepts inspired by the literature on mixed methods research [22–24]: (a) synthesis methods, (b) sequence of data synthesis, and (c) integration of data and synthesis results.

- *Synthesis methods*: Synthesis consists of the stage of a review when the evidence extracted from the individual sources is brought together [13]. The synthesis method was identified from information provided in the Methods and Results sections. In line with the literature on mixed methods research, the synthesis methods were classified as quantitative or qualitative based on the process and output generated. A synthesis method was considered quantitative when the main results on specific variables across included studies were summarized or combined [25]. Quantitative output is based on numerical values of variables, which are typically produced using validated and reliable checklists and scales and are used to produce numerical data and summaries (such as frequency, mean, confidence interval, and standard error) and conduct statistical analyses [26]. Conversely, a synthesis method was considered qualitative when it summarized or interpreted data to generate outputs such as themes, concepts, frameworks, or theories (inter-related concepts).
  The distinction between qualitative and quantitative synthesis methods was clear in most cases. However, some synthesis methods required further discussion between the reviewers. For example, in this review of reviews, a distinction between qualitative and quantitative content analysis was made. Content analysis described in Neuendorf [27] and Krippendorff [28] was considered quantitative synthesis method because the coded categories are reliable variables and values allowing descriptive and analytical statistics. This method was developed over a century ago and is defined “as the systematic, objective, quantitative analysis of message characteristics” [27]. In contrast, qualitative content analysis produces themes and subthemes that are qualitative in nature [29]. Also, in some SMSRs, the synthesis methods were not considered quantitative even if numbers were provided in the results. For example, some presented a table of frequencies of the number of studies for each theme identified from a thematic synthesis. The synthesis was considered qualitative since the main outputs were themes, while the numbers did not provide a combined estimate of a specific variable. Moreover, some synthesis methods are not exclusively qualitative or quantitative. For example, configurational comparative method has been considered simultaneously quantitative and qualitative by the developers [30]. In this review of reviews, this method was considered quantitative because it relies on logical inferences (Boolean algebra) and aims to reduce cases to a series of variables. Another synthesis method requiring discussion was vote counting that is considered quantitative in the literature [31]. In this review of reviews, vote counting was considered qualitative when the results were only used for descriptive purpose.
  Tables 4 and 5 present a list of quantitative and qualitative synthesis methods found in the literature [13, 32–34]. When there was a discrepancy between the method described and the method used, the information from the latter was considered during the analysis. For example, some reviews described meta-analysis in the Methods section yet indicated in the Results section that the data were too heterogeneous to be combined quantitatively and a narrative analysis was, thus, used. In this case, the synthesis was considered as qualitative.

**Table 4:** Quantitative synthesis methods

Synthesis method	Aim	Description
Bayesian synthesis [53]	To measure the likelihood of different values for parameters of interest.	Incorporates prior distributions of unknown parameter values that are then updated by deriving posterior probability distributions generated through statistical analysis of the estimates.
Case survey [54, 55]	To identify and statistically test patterns across individual case studies.	Converts qualitative cases into quantitative variables by extracting data using a same set of closed-ended questions. The answers to these questions are then aggregated to establish frequency of occurrence (that can be further statistically analyzed, as appropriate).
Configurational comparative method [56]	To build or test theories and assumptions by identifying configurations of causal conditions, i.e., combination of conditions (independent variables) that are necessary and/or sufficient for a given outcome (dependent variable).	Consists in a comparative case-oriented research approach that uses Boolean algebra to generate configurations between conditions and outcomes across cases.
Cross-design synthesis [57]	To combine results from quantitative studies with complementary designs (e.g., RCT and observational studies).	Involves an in-depth assessment of key biases of each study, an adjustment of each study’s results based on the identified biases and the development of a model for combining the results within and across designs.
Meta-analysis [58]	To obtain a single summarized “effect size.”	Uses statistical methods for combining results of studies into a weighted average of point estimates.
Meta-regression [59]	To relate the size of effect to one or more characteristics of the included studies (to explore sources of heterogeneity across included studies).	Uses a combination of meta-analytic and regression principles.
Meta-summary [60]	To quantitatively aggregate qualitative findings.	Consists of extraction, grouping, abstraction, and formatting of findings and the calculation of frequency and intensity effect sizes.
Quantitative content analysis [27, 28]	To transform qualitative data into few variables (numerical value) for statistical analysis.	Categorizes data and provides statistical description of the categories.
Vote counting [61]	To calculate the frequencies of categories of results across included studies.	The included studies are sorted into three categories (negative significant, positive significant, and statistically insignificant), and the number of studies for each category is calculated. The category with the most studies is the “winner.”

**Table 5:** Qualitative synthesis methods

Synthesis method	Aim	Description
Critical interpretive synthesis [62]	To build a theory from the synthesis of a diverse body of evidence.	Adapted the strategies of meta-ethnography (reciprocal translational analysis, lines-of-argument synthesis, and refutational syntheses) for qualitative and quantitative evidence.
Framework synthesis [63]	To produce a new framework based on a priori and new themes.	Consists of analyzing data using an a priori framework, creating new themes by performing thematic synthesis, and producing a new framework.
Grouping and clustering [44]	To describe included studies.	Summarizes and organizes included studies into groups (categories).
Meta-ethnography [64]	To build a theory from the synthesis of qualitative studies.	Uses three main strategies: translating the concepts from studies into one another (reciprocal translational analysis), exploring and explaining contradictions between studies (refutational synthesis), and linking constructs and building a picture of the whole from studies (lines-of-argument synthesis).
Meta-narrative synthesis [65]	To make sense of complex and conflicting findings by unfolding the storyline of research traditions.	Maps research traditions and consider how they have been conceptualized, theorized, and empirically studied over time.
Meta-synthesis [66]	To understand a phenomenon of interest across qualitative studies.	Uses hermeneutic (portraying individual constructions) and dialectic (comparing and contrasting the constructions) approaches.
Narrative synthesis [44]	To summarize and explain the findings of included studies.	Adopts a textual approach to the process of synthesis and follows four elements: develop a theory of how the intervention works, why, and for whom; develop a preliminary synthesis; explore relationships within and between studies; and assess the robustness of the synthesis.
Qualitative content analysis [29]	To understand a phenomenon of interest by focusing on the manifest (patent) content or contextual meaning of text.	Uses an analytical coding process to organize content of textual data into fewer content categories.
Realist synthesis [67, 68]	To unpack how interventions work in particular contexts through theoretical explanation (middle-range theory).	Uses theory-driven context-mechanism-outcome configurations, demi-regularities, and abduction (hunches).
Textual description [44]	To describe included studies.	Provides a descriptive paragraph of each study.
Textual narrative synthesis [69]	To describe included studies.	Arranges studies into homogeneous groups and compares similarities and differences across studies.
Thematic synthesis [70]	To identify and develop themes across included studies.	Uses line-by-line coding, develops descriptive themes, and generates analytical themes. This might lead to propose a conceptual framework.
  Within each review, one or several synthesis methods could be used. The synthesis process could be either qualitative (i.e., used one or several qualitative synthesis methods to analyze the included studies), quantitative (i.e., used one or several quantitative synthesis methods to analyze the included studies), or mixed (i.e., used both qualitative and quantitative synthesis methods to analyze the included studies).
- *Sequence*: In the literature on mixed methods research, a sequence refers to a temporal relationship between qualitative and quantitative methods of data collection and analysis [15]. In this review of reviews, the sequence of the analysis was determined based on the number of phases of synthesis and whether the results of one phase informed the synthesis of a subsequent phase. For example, a qualitative synthesis of qualitative studies is done first to identify the components of an intervention (phase 1). Then, the quantitative studies are analyzed to quantify the effect of each component (phase 2). In this case, we considered there was a sequence because the results of the qualitative synthesis informed the quantitative synthesis.
- *Integration*: In the literature on mixed methods research, integration is defined as the process of bringing (mixing) qualitative and quantitative approaches together and can be achieved at the level of the design (e.g., sequential and convergent designs), the methods (data collection and analysis), and the interpretation and reporting [35, 36]. In this review of reviews, we adapted these levels of integration: (1) data, i.e., all evidence analyzed using a same synthesis method, (2) results of syntheses, i.e., the results of the synthesis of qualitative and quantitative evidence are compared or combined, (3) interpretation, i.e., the discussion of the results of the synthesis of qualitative and quantitative evidence, and (4) design.

## Results

### Description of included reviews

**Fig. 1:**
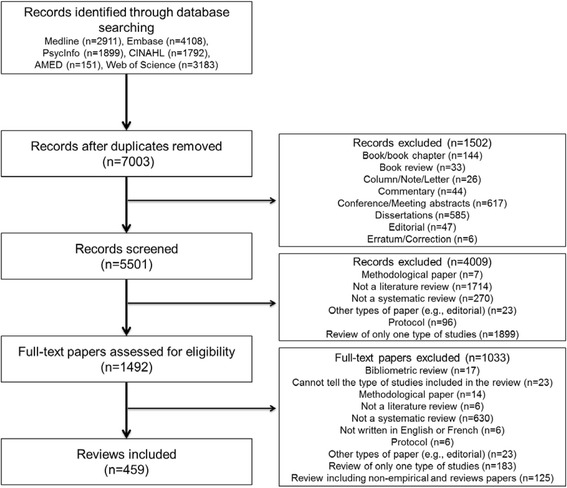
Flowchart

**Fig. 2:**
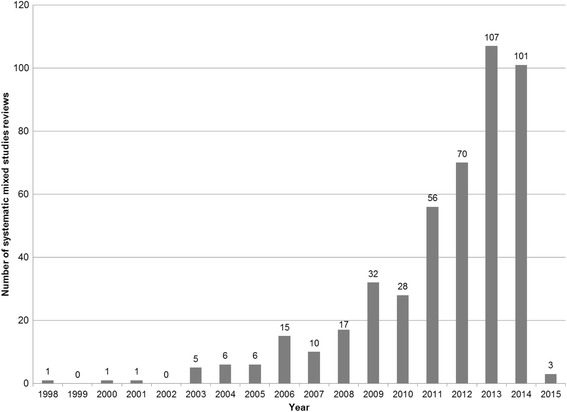
Number of systematic mixed studies reviews published per year

The bibliographic database search yielded 7003 records of which 459 SMSRs were included in this review of reviews (Fig. 1). As seen in Fig. 2, there has been an exponential progression of the number of publications per year, especially since 2010. In over a decade, the number has passed from nearly 10 per year to more than 100. The topics of the SMSRs were mainly in health and varied widely, from health care to public health. Some were on information sciences, management, education, and research. The first authors of the SMSRs came from 28 different countries. The countries producing the most SMSRs are England (*n* = 179), Australia (*n* = 71), the USA (*n* = 53), Canada (*n* = 45), and the Netherlands (*n* = 20).

Several labels were used to name this type of review, with the most common being “systematic review” (*n* = 277), followed by “literature review” (*n* = 39), “integrative review” (*n* = 35), and “mixed methods reviews” (*n* = 24). Among those using the term systematic review, a small number specified in the title that they combined different types of evidence: “mixed systematic review” (*n* = 2), and “systematic review of quantitative and qualitative” data, evidence, literature, research, or studies (*n* = 23).

The number of studies included in the SMSRs ranged from 2 to 295 (mean = 29; SD = 33). The majority of SMSRs included qualitative and quantitative studies (*n* = 249) or qualitative, quantitative, and mixed methods studies (*n* = 200). Few included only quantitative and mixed methods studies (*n* = 8) or only qualitative and mixed methods studies (*n* = 2).

Only 24% (*n* = 110) of included reviews provided a clear rationale for combining quantitative and qualitative evidence. Authors described various reasons for performing SMSRs that fall into the following eight categories: (a) nature of the literature on a topic—to adapt the review method because of the limited evidence on the topic or absence of RCTs, (b) complexity of the phenomenon—to address a complex and multifaceted phenomenon, (c) broad coverage—to provide broader perspective and cover a wide range of purposes, (d) comprehensiveness—to provide a complete picture and deduce the maximum information from the literature, (e) thorough understanding—to gain better and detailed understanding of a phenomenon, (f) complementarity—to address different review questions (e.g., why and how) and complement the strengths and limitations of quantitative and qualitative evidence, (g) corroboration—to strengthen and support the results through triangulation, and (h) practical implication—to provide more meaningful and relevant evidence for practice.

Only 39% (*n* = 179) of included reviews provided a full description of the synthesis method(s) with methodological references. The remainder provided information without reference (*n* = 149), simply mentioned (labelled) the synthesis method used (*n* = 41), or did not provide information about the synthesis (*n* = 90). A variety of synthesis methods were used in the included reviews. Among the SMSRs that provided information on the synthesis methods, the most common method mentioned was thematic synthesis (*n* = 129), followed by narrative synthesis (*n* = 64), narrative summary (*n* = 30), categorization/grouping (*n* = 20), content analysis (*n* = 30), meta-synthesis (*n* = 25), meta-analysis (*n* = 27), narrative analysis (*n* = 11), meta-ethnography (*n* = 9), textual narrative (*n* = 7), framework synthesis (*n* = 7), and realist synthesis (*n* = 6).

### Synthesis of results

**Fig. 3:**
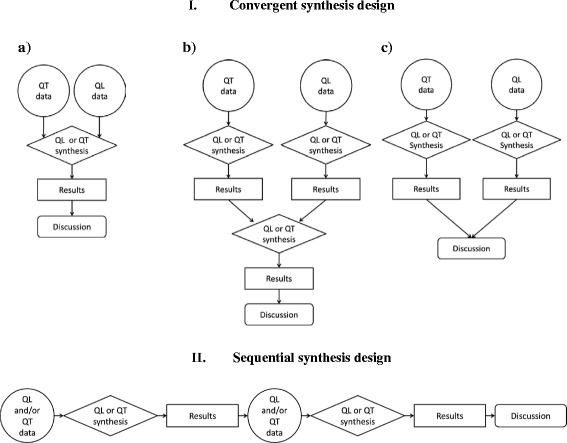
Typology of synthesis design in mixed studies reviews. *QL* qualitative, *QT* quantitative. **a** Data-based convergent synthesis design. **b** Results-based convergent synthesis design. **c** Parallel-results convergent synthesis design

Based on the sequence and integration concepts, two main types of synthesis designs were identified (Fig. 3): convergent and sequential synthesis designs. Within the convergent synthesis design, three subtypes were found: data-based, results-based, and parallel-results convergent synthesis designs. These synthesis designs were cross tabulated with the three types of synthesis methods (qualitative, quantitative, and mixed). This led to a total of 12 possible synthesis strategies that are represented in Table 6. Reviews were found for eight of these possibilities.

**Table 6:** Percentages of systematic mixed studies reviews among the 12 synthesis strategies (*n* = 459)

	Sequence and integration				
Synthesis	Convergent synthesis design			Sequential synthesis design	Total
	Data-based	Results-based	Parallel-results		
Qualitative	69.5%	6.3%	12.0%	2.6%	90.4%
Quantitative	0.2%	0%	0%	0%	0.2%
Mixed	0%	2.2%	5.2%	2.0%	9.4%
Total	69.7%	8.5%	17.2%	4.6%	100%

I. Convergent synthesis design: In this design, the quantitative and qualitative evidence is collected and analyzed during the same phase of the research process in a parallel or a complementary manner. Three subtypes were identified based on where the integration occurred.
  - Data-based convergent synthesis design (Fig. 3a): This design was the most common type of synthesis design (Table 6). In this design, all included studies are analyzed using the same synthesis method and results are presented together. Since only one synthesis method is used for all evidence, data transformation is involved (e.g., qualitative data transformed into numerical values or quantitative data are transformed into categories/themes). This design usually addressed one review question. Among the SMSRs in this design, three main objectives were found. The first category sought to describe the findings of the included studies, and the synthesis methods ranged from summarizing each study to grouping main findings. The review questions were generally broad (similar to a scoping review) such as what is known about a specific topic. The second category consisted of SMSRs that sought to identify and define main concepts or themes using a synthesis method such as qualitative content analysis or thematic synthesis. The review questions were generally more specific such as identifying the main barriers and facilitators to the implementation of a program or types of impact. The third category included SMSRs that aimed to establish relationships between the concepts and themes identified from the included studies or to provide a framework/theory.
  - Results-based convergent synthesis design (Fig. 3b): Nearly 9% of SMSRs were classified in this synthesis design (Table 6). In this design, the qualitative and quantitative evidence is analyzed and presented separately but integrated using another synthesis method. The integration could consist of comparing or juxtaposing the findings of qualitative and quantitative evidence using tables and matrices or reanalyzing evidence in light of the results of both syntheses. For example, Harden and Thomas [6] suggest performing a quantitative synthesis (e.g., meta-analysis) of trials and a qualitative synthesis of studies of people’s views (e.g., thematic synthesis). Then, the results of both syntheses are combined in a third synthesis. This type of design usually addresses an overall review question with subquestions.
  - Parallel-results convergent design (Fig. 3c): A little over 17% of reviews were classified in this design (Table 6). In this design, qualitative and quantitative evidence is analyzed and presented separately. The integration occurs during the interpretation of results in the Discussion section. Some of these SMSRs included two or more complementary review questions. For example, health technology assessments evaluate several dimensions such as clinical effectiveness, cost-effectiveness, and acceptability of an intervention. The evidence of each dimension is reviewed separately and brought together in the discussion and recommendations.
II. Sequential synthesis design (Fig. 3): This design was found in less than 5% of the reviews (Table 6). It involves a two-phase approach where the data collection and analysis of one type of evidence occur after and are informed by the collection and analysis of the other type. This design usually addressed one overall review question with subquestions and both syntheses complemented each other. For example, in a review aiming at identifying the obstacles to treatment adherence, the qualitative synthesis provided a list of barriers and the quantitative synthesis reported the prevalence of these barriers and knowledge gaps (barriers for which prevalence was not estimated) [37].

## Discussion

The number of published SMSRs has considerably increased in the past few years. In a previous review of reviews in 2006, Pluye et al. [9] identified only 17 SMSRs. This shows that there is an increasing interest for this type of review and warrants the need for more methodological development in this field.

In accordance with the literature on mixed methods research, two main types of synthesis designs were identified in this review of reviews: convergent and sequential synthesis designs. Three subtypes of convergent synthesis were found: data-based convergent, results-based, and parallel-results convergent synthesis designs. The data-based convergent design was more frequently used probably because it is easier to perform, especially for a descriptive purpose. The other synthesis designs might be more complex but could allow for greater analytical depth and breadth of the literature on a specific topic. Also, focusing the analysis on the concepts of convergent and sequential designs allowed us to clarify and refine their definitions. Considering that the focus of the analysis was the synthesis process in SMSRs, the literature on process studies especially in the fields of management provides insight into these concepts. First, in line with Langley et al. [38], the convergent design can be defined as a process of gradual, successive, and constant refinements of synthesis and interpretation of the qualitative and quantitative evidence. Researchers are working forward in a non-linear manner guided by a cognitive representation of new data-based synthesis or results-based synthesis or interpretation of results to be created. Second, in line with Van de Ven [39], a sequential synthesis design can be defined, according to a developmental perspective (phase 1 informing phase 2; phase 2 building on the results of phase 1), as a change of focus at the level of data or synthesis over time and as a cognitive transition into a new phase (e.g., from qualitative to quantitative or from quantitative to qualitative).

The synthesis designs found in this review of reviews reflect those suggested by Sandelowski et al. [8] (see Table 1) who used the terms *segregated*, which can be similar to results-based and parallel-results convergent synthesis designs, *integrated*, which is comparable to data-based convergent synthesis design, and *contingent* designs, which could be considered as a form of sequential design. In this review of reviews, we used the mixed methods concepts and terminology because they account for the integration that may be present at the level of data, results, interpretation, or design.

As in Heyvaert et al. [22], the concepts found in the literature on mixed methods research to define the synthesis designs were used; yet, the definition of the synthesis method and integration concepts was somewhat different. In Heyvaert et al. [22], they focused on the relative importance of methods, i.e., whether the qualitative or the quantitative method was dominant or of equal status. This was not done in this review of reviews because measuring or documenting the dominance of a method is difficult given the influences of multiple factors (power, resources, expertise, time, training, and worldviews of each research team member, among other factors). Also, in Heyvaert et al. [22], they considered that integration could be partial (i.e., part of the qualitative and quantitative studies are involved separately in some or all stages) or full (i.e., all the qualitative and quantitative studies are involved in all the stages). In this review of reviews, the focus was put on where the integration occurred. Therefore, this review of reviews resulted in respectively four and three types of synthesis designs and methods, which led to propose 12 synthesis strategies, as compared to 18 in Heyvaert et al. [22].

In Frantzen and Fetters [40], three main types of convergent designs are suggested (see Table 1). Similarly, this review of reviews also found qualitative, quantitative, or mixed convergent synthesis design types. However, no distinction was made during the analysis between SMSRs including only qualitative and quantitative studies (basic type) and those also including mixed methods studies (advanced type) because this review of reviews aimed at defining ideal types of synthesis designs. The paper written by Frantzen and Fetters [40] went into deeper analysis of convergent design to provide detailed information on the steps to follow to integrate qualitative, quantitative, and mixed methods studies.

Some SMSRs using sequential synthesis design were found in our sample of reviews. Pluye and Hong [10] suggested using the sequential exploratory or explanatory designs. In the exploratory sequential design, a qualitative synthesis is performed first and results inform the subsequent quantitative synthesis. Conversely, in an explanatory sequential design, the quantitative synthesis is done first and informs the subsequent qualitative synthesis. In this review of reviews, the sequence was defined as the results of one phase informing the other (not limited to the order of the syntheses) and no review was classified as sequential explanatory. In addition, 12 SMSRs performing only qualitative syntheses were found and could not be classified as exploratory or explanatory. For the sake of parsimony, we did not make a distinction between exploratory and explanatory sequential synthesis designs.

### Implications for conducting and reporting mixed studies reviews

In light of this review of reviews and the literature on mixed methods research, four complementary key recommendations can be made regarding the title, justification, synthesis methods, and the integration of qualitative and quantitative data.

First, researchers should explicitly state in the title that the review included qualitative and quantitative evidence. Various terms are used to designate this type of review. Some SMSRs used the term “mixed” such as mixed systematic review, mixed methods review, mixed research synthesis, or mixed studies review. The term mixed has been used in the mixed methods literature to designate primary research designs combining qualitative and quantitative approaches [23]. In the field of review, mixing qualitative and quantitative evidence can be seen at two levels: study level and synthesis level [22]. Pluye et al. [9] suggested “mixed studies review” referring to a review of studies of different designs. This name focuses on the study level and does not prescribe a specific synthesis method. Others have suggested labelling this type of review as mixed methods review [6, 22] wherein mixing occurs at both the level of the study and the synthesis. Another popular term is integrative review proposed by Whittemore and Knafl [5]. Integrative review is described as a type of literature review to synthesize the results of research, methods, or theories using a narrative analysis [41]. Currently, all these terms are used interchangeably without a clear distinction [40].

Second, researchers should provide a clear justification for performing a SMSR and describe the synthesis design used. In this review of reviews, this information was found in only 24% of the SMSRs. This lack of justification for using qualitative and quantitative evidence is also found in the literature on mixed methods research [42]. The rationale will influence the review questions and the choice of the synthesis design. For example, if quantitative and qualitative evidence is used for corroboration purpose, the convergent synthesis design may be more relevant. On the other hand, when they are used in complementarity such as using the quantitative studies to generalize qualitative findings or using qualitative studies to interpret, explain, or provide more insight to some quantitative findings, the sequential synthesis design may be more appropriate.

Third, results of this review of reviews suggest a need to recommend that researchers describe their synthesis methods and cite methodological references. Only 39% of the SMSRs provided a full description of the synthesis methods with methodological references. Various synthesis methods have been developed over the past decade [13, 32, 33, 43]. Meta-analysis is the best known synthesis method to aggregate findings in reviews, especially for clinical effectiveness questions. However, when this method is not possible, researchers tend to omit describing the synthesis. Researchers should avoid limiting the description to what was not done such as using the sentence “because of the heterogeneity of studies, no meta-analysis was performed and data were analyzed narratively.” The term “narrative” can be confusing since it is often used differently by different authors. In some SMSRs, narrative analysis corresponded to summarizing each included study. In others, it consisted in grouping the different findings of included studies into main categories and summarizing the evidence of each category. Still, others followed Popay et al.’s [44] four main elements for narrative synthesis (i.e., develop a theoretical model, preliminary synthesis, relationship, and assess robustness). Hence, in addition to naming the synthesis method, we recommend that reviews should provide a clear description of what was done to synthesize the data and add methodological references. This will improve transparency of the review process, which is an essential quality of systematic reviews.

Fourth, researchers should describe how the data were integrated and discuss the insight gained from this process. Integration is an inherent component of mixed methods research [15], and careful attention must be paid to how integration is done and reported to enhance the value of a review. The synthesis designs outline that can provide guidance on how to integrate data (Fig. 3). Also, the discussion should include more than a simple wrap-up of results. It should clearly reflect on the added value and insight gained of combining qualitative and quantitative evidence into a review.

### Limitations

The search strategy used was not comprehensive; thus, not all SMSRs were identified in this review of reviews. Indeed, the search was limited to six databases mainly in health and no hand searching was performed. As this review of reviews deals with methods, citation tracking of included SMSRs would not have provided additional relevant references. Nonetheless, our sample of included SMSRs was large (*n* = 459) and sufficient to achieve the aim of this review of reviews.

To ensure a manageable sample size, selection of included reviews was limited to peer-reviewed journal articles. We acknowledge that the sample of included reviews might not include some innovative developments in this field, given that some recent SMSRs may be reported in other types of publications (e.g., conference abstracts or gray literature).

Finally, the synthesis methods were not classified as aggregative and configurative [45, 46]. As mentioned in Gough et al. [45], some configurative synthesis can include aggregative component and vice versa. To avoid this confusion, the terms qualitative and quantitative synthesis methods were preferred. Moreover, these terms were used to align with the mixed methods research terminology. Yet, as discussed in the Methods section, the interpretation of some synthesis methods used in this review of reviews can be debatable.

## Conclusions

The field of SMSR is still young, though rapidly evolving. This review of reviews focused on how the qualitative and quantitative evidence is synthesized and integrated in SMSRs and suggested a typology of synthesis designs. The analysis of this literature also highlighted a lack of transparency in reporting how data were synthesized and a lack of consistency in the terminology used. Some avenues for future research can be suggested. First, there is a need to reach consensus on the terminology and definition of SMSRs. Moreover, given the wide range of approaches to synthesis, clear guidance and training are required regarding which synthesis methods to use and when and how they should be used. Also, future research should focus on the development, validation, and reliability testing of quality appraisal criteria and standards of high-quality SMSRs. Finally, an adapted PRISMA statement for reporting SMSRs should be developed to help advance the field.

## Abbreviations

PRISMA: Preferred Reporting Items for Systematic Reviews and Meta-Analyses
QL: Qualitative
QT: Quantitative
RCT: Randomized controlled trial
SMSR: Systematic mixed studies review
